# An overview of image registration for aligning mass spectrometry imaging with clinically relevant imaging modalities

**DOI:** 10.1016/j.jmsacl.2021.12.006

**Published:** 2021-12-18

**Authors:** Benjamin Balluff, Ron M.A. Heeren, Alan M. Race

**Affiliations:** a Maastricht MultiModal Molecular Imaging Institute (M4i), Maastricht University, Maastricht, the Netherlands; b Institute of Medical Bioinformatics and Biostatistics, Philipps University of Marburg, Marburg, Germany

**Keywords:** 2D, two dimensional, 3D, three dimensional, CT, computed tomography, DESI, desorption electrospray ionisation, H&E, haematoxylin & eosin, ICP, inductively coupled plasma, IHC, immunohistochemistry, LA-ICP-MS, laser ablation inductively coupled plasma mass spectrometry, LMD, laser microdissection, MALDI, matrix-assisted laser desorption/ionisation, MSI, mass spectrometry imaging, MRI, magnetic resonance imaging, PCA, principal component analysis, PET, positron emission tomography, RGB, red–green-blue, SIMS, secondary ion mass spectrometry, tSNE, t-distributed stochastic neighbourhood embedding, UMAP, uniform manifold approximation and projection, Mass spectrometry imaging, Image registration, Image integration, Histology, Magnetic resonance imaging

## Abstract

Mass spectrometry imaging (MSI) is used in many aspects of clinical research, including pharmacokinetics, toxicology, personalised medicine, and surgical decision-making. Maximising its potential requires the spatial integration of MSI images with imaging data from existing clinical imaging modalities, such as histology and MRI. To ensure that the information is properly integrated, all contributing images must be accurately aligned. This process is called image registration and is the focus of this review. In light of the ever-increasing spatial resolution of MSI instrumentation and a diversification of multi-modal MSI studies (e.g., spatial omics, 3D-MSI), the accuracy, versatility, and precision of image registration must increase accordingly. We review the application of image registration to align MSI data with different clinically relevant *ex vivo and in vivo* imaging techniques. Based on this, we identify steps in the current image registration processes where there is potential for improvement. Finally, we propose a roadmap for community efforts to address these challenges in order to increase registration quality and help MSI to fully exploit its multi-modal potential.

## Highlights

• Mass spectrometry imaging (MSI) is a powerful molecular imaging technique.
• Integration with other imaging modalities is essential in clinical MSI.
• Image integration is performed by image registration techniques.
• Technical potential of image registration in MSI has not been fully exploited.
• Roadmap proposed to improve registration accuracy.

## Graphical abstract

**Figure:**
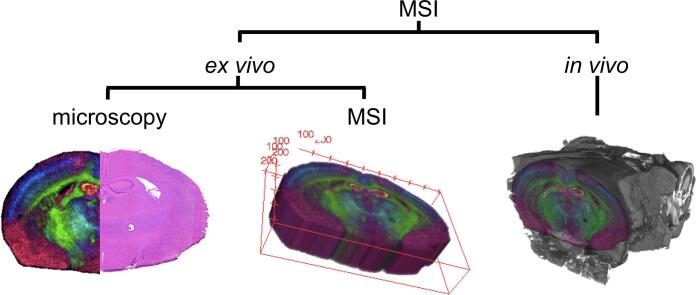

## Introduction

Mass spectrometry imaging (MSI) is a molecular imaging technology to visualise the spatial distributions and abundance of molecules or elements on a micrometre or nanometre scale. Matrix-assisted laser desorption/ionisation (MALDI) and desorption electrospray ionisation (DESI) MSI have become particularly useful for the study of clinical tissue specimens since they allow the soft ionisation of different molecular classes (including larger biomolecules, such as proteins) at spatial resolutions that are approaching that of the clinical gold standard (microscopic evaluation by a pathologist) [1], [2].

**Fig. 1:**
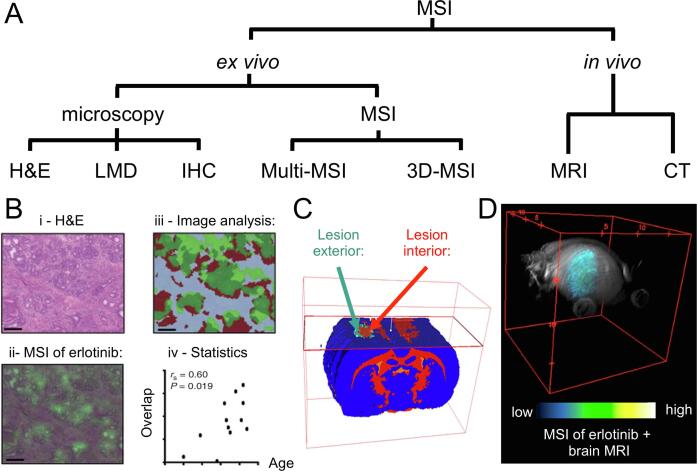
(A) Most common spatial integrations of mass spectrometry imaging (MSI) with clinically relevant *ex vivo* and *in vivo* modalities. The spatial integration is facilitated by image registration workflows, which need to be adapted to the characteristics of each multi-modal combination. (B) MSI images of the anti-cancer drug erlotinib in mouse models of pancreatic cancer (ii) were integrated with the corresponding histological images (H&E) obtained by optical microscopy (i). Quantifying the spatial overlap of erlotinib (green) with the glandular structures (red) by digital image analysis algorithms (iii), allowed observation of the life-prolonging effect of erlotinib on the mice (iv). Adapted from [5]. Copyright 2016, American Association for Cancer Research. (C) 3D-MSI of lipids and unsupervised clustering were used to reveal the volumetric, stratified, and molecular expansion of traumatic brain injury in rats after controlled cortical impact [9]. Copyright 2018 American Chemical Society. (D) 3D-MSI facilitates the integration with 3D *in vivo* imaging data such as provided by MRI. Here, 3D-MSI was performed to obtain the volumetric distribution of the anti-cancer drug erlotinib in glioblastoma mouse brain models and nonlinearly coregistered to the corresponding 3D-MRI images. Adapted with permission from [10]. Copyright 2019 American Chemical Society. Abbreviations used: mass spectrometry imaging, MSI; haematoxylin and eosin staining, H&E; laser microdissection, LMD; immunohistochemistry, IHC; magnetic resonance imaging, MRI.

For this reason, most clinically motivated MALDI and DESI MSI studies have included histological information in order to link the spatially resolved molecular profiles with the underlying cell [3], [4]. Grüner *et al*., for instance, were only able to measure the age-prolonging effect of the anti-cancer drug erlotinib (detected by MSI) when quantifying the spatial overlap of its distribution with the glandular structures in the pancreas (detected by optical microscopy) (Fig. 1B) [5]. Similarly, Prade *et al*. have used immunohistochemistry to spatially link clinically relevant molecular tumour cell phenotypes (Her2 and cytokeratin) with their distinct metabolic pathways, as detected by MSI [6].

In DESI and MALDI the set of observable molecular classes depends heavily on the sample preparation used, such as the choice of solvent or matrix, respectively. To take advantage of this, multiple MSI datasets capturing different molecular classes can be spatially integrated to obtain a more comprehensive molecular description of a sample, as shown by Patterson and co-workers [7].

Spatial alignment of MSI images is also imperatively performed in 3D-DESI and 3D-MALDI-MSI, when equidistant consecutive sections are obtained for a volumetric reconstruction of the sample [8]. Mallah *et al*. for instance, demonstrated how 3D-MSI and volumetric reconstruction can be used to molecularly characterize the spatial expansion of traumatic brain injury (Fig. 1C) [9].

A volumetric reconstructed 3D-MSI dataset is usually the basis for further integration with other clinically relevant imaging techniques, such as magnetic resonance imaging (MRI). Using this approach, Abdelmoula *et al*. were able to relate the volumetric distribution of the anti-cancer drug erlotinib in MRI images of glioblastoma mouse brain models (Fig. 1D) [10].

**Fig. 2:**
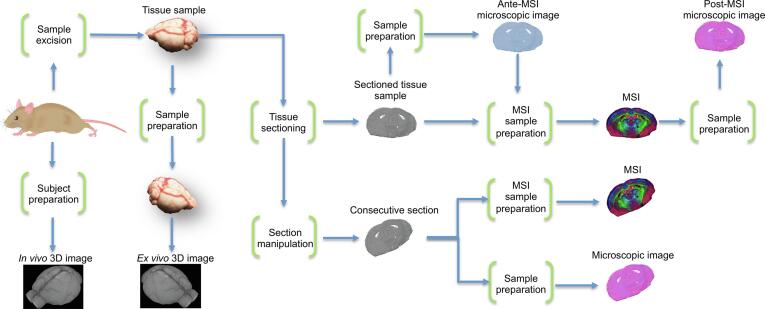
General overview of experimental workflow, which summarizes the most commonly-employed modalities involved in multimodal MSI studies. Sample preparation and manipulation steps that can cause changes between the resulting images – excluding differences in the images that are caused by the technologies themselves – are shown as green brackets. These alterations can be a change in the general orientation of the sample, but also local deformations. Image registration between two images has to account for all effects caused by the sample preparation/manipulation steps that were traversed on the path between two images. Abbreviations used: mass spectrometry imaging, MSI.

In all of these examples, the spatial overlay of the multi-modal images, which was enabled by *image registration* techniques, was crucial to the success of the study. Image registration is a broad field of research, with applications in medical imaging [11], remote sensing [12] and astronomy [13]. As the physical properties of the imaging systems can differ between fields (consider magnetic resonance imaging and microscopy, for example) in the same way that the target can differ (for example a human head in medical imaging and stars in astronomy) there is no single technique that can be applied to all image registration problems. For this reason, methods have been developed and optimised for aligning the specific style of data generated in mass spectrometry imaging with complementary techniques. Many of these newly developed methods build upon work in other fields, especially those in medical imaging, as discussed in the following sections. The choice of the image registration strategy thereby depends on the expected image alterations (introduced by the experimental workflow, see Fig. 2) and the clinical imaging modalities involved (Fig. 1A).

Until recently, most MSI studies have used simple image registration techniques, which resulted in spatial alignment errors well below the MSI pixel size [14]. As the registration process aims to find a transformation which, when applied, ensures all features within one image overlap exactly with the corresponding features in the second image, spatial alignment errors refer to any deviation between these features following the registration and transformation process. This is discussed in more detail in the Section Improving and measuring accuracy. With advances in instrumentation and sample preparation enabling ever-smaller pixel sizes in MSI experiments, precise registration has become one of the bottlenecks to fully exploit high-resolution MSI data for single cell or subcellular investigations [15], [16].

Consequently, image registration should become a focus of the MSI community in order to develop new solutions for spatially accurate integration of different modalities with MSI. In this review, we assess the current state of image registration in different multi-modal MSI applications with respect to their aims, challenges, and technical solutions. Based on this, we propose a road map for the community to address the current challenges of image registration in MSI.

## Image registration and transformation

### Mass spectrometry imaging workflow

Image registration forms the basis for the spatial alignment of two images that have not been acquired under the same conditions. These different conditions can be, but are not limited to, a change in orientation of the sample, a change in spatial resolution, or the use of different modalities leading to the visualisation of different information via potentially different contrast mechanisms.

In MSI studies, the many sample manipulation and preparation steps needed for MSI and the other involved modalities are likely to cause additional local or global deformations of the tissue and/or modification of its molecular content, which can lead to additional differences in the images. This can be due to the exposure of the sample to thermic (e.g., antigen-retrieval for peptide imaging or immunohistochemistry), chemical (e.g., washes to remove salts and/or lipids for peptide imaging), and mechanical (e.g., sectioning) manipulations during the experimental workflow. Image registration must account for the technological nature of the involved imaging modalities as well as any sample preparation and manipulation steps that lie between the modalities to be linked (Fig. 2). As a consequence of this, although the use of the same tissue section for different modalities can reduce the number of sample preparation/manipulations steps, image registration is still required.

### Image registration

**Fig. 3:**
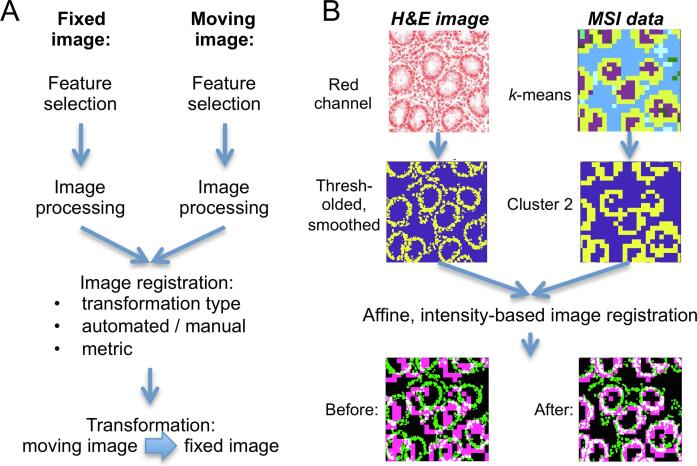
(A) General image registration workflow for the alignment of the moving image to fit the fixed image. (B) An example workflow is shown with the aim to register MSI data to the corresponding histological image (H&E). Appropriate and matching features are selected and enhanced in both images using *k*-means clustering and image processing in the MSI and histological image, respectively. Intensity-based automatic registration, optimising a measure of similarity (in this case, mutual information), results in a geometric transformation, which, once applied, transforms the moving MSI image (Before) to fit the fixed image (After). Abbreviations used: mass spectrometry imaging, MSI; haematoxylin and eosin staining, H&E.

While image registration includes a family of techniques, most follow the same workflow (Fig. 3A). What they have in common is that the user needs to define which representation of the data should be used to align the two imaging datasets. This representation could be a selected channel of the image (for example, any of the RGB channels for optical images), a transformed representation of the image (such as a dichotomised intensity image), or corresponding landmark points in both images. Image processing can optionally enhance these representative images where, for instance, morphological structures of the tissue or its contours are detected and emphasized (Fig. 3B, left).

In MSI, due to the multichannel nature of mass spectrometric data, either single channels can be selected and optionally processed, or multivariate algorithms can be used to squeeze the multivariate information into single-channel representative images. These multivariate algorithms can be unsupervised or supervised [17]. As an example of the use of unsupervised methods, dimensionality reduction (e.g., by principal component analysis, PCA) can be used to create component images where a pixel’s value carries the scores for a selected component and can hence be interpreted as intensity. In contrast, clustering methods (also unsupervised) can be used to assign every pixel of the MS image to a cluster, with the consequence that a pixel’s value becomes categorical. Likewise, supervised classification methods can be used to create single-channel representative images with categorical values from multivariate MSI data [18].

Once this step has been reached, one must decide which of the two images shall be the target (*fixed*) image and which will be the transformed (*moving*) image. Convention is that the image with the higher spatial resolution takes the role of the fixed image (Fig. 3B). From this moment on, different image registration techniques can be distinguished based on the type of transformation, the level of interactivity, the modalities involved (mono- vs. multi-modal), and the (spatial) dimensionality of the registration task (2D vs. 3D) [19].

**Fig. 4:**
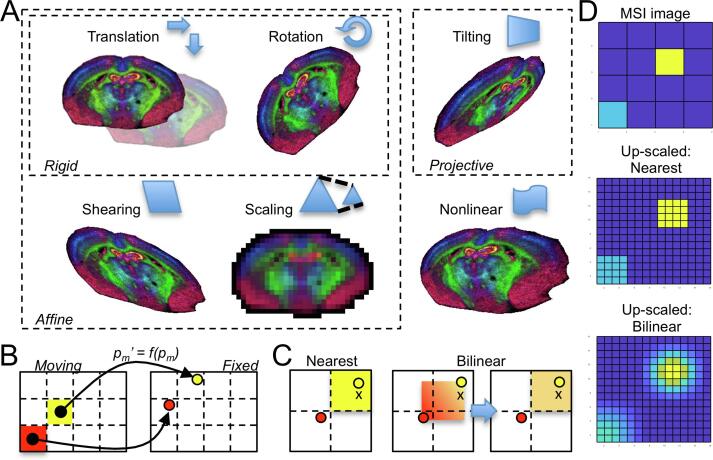
(A) Examples of geometric transformations are demonstrated on a MSI dataset of a coronal mouse brain section using rigid transformation (translation, rotation), affine transformation (scaling, shearing), projective transformation (tilting), and nonlinear transformation. (B) The actual transformation of the moving image is achieved by applying a function *f* to all pixels of the moving space (*p_m_*) in order to obtain their position in the fixed space (*p_m_*′). (C) The fact that transformed points do not precisely match the naturally numbered raster grid positions of the fixed image requires the application of interpolation methods to estimate the intensities at the pixel centres (“x”) of the fixed image. “Nearest” (left) assigns a pixel the intensity of the closest *p_m_*′. “Bilinear” (right) assigns the intensity to the current pixel by interpolating the intensities of the surrounding *p_m_*′ in a two dimensional fashion.

The type of transformation constitutes the most important decision since it defines the possible geometric manipulations that can be applied to the moving image. Transformation models can be rigid, non-rigid (affine), projective, or nonlinear (Fig. 4A). The first three belong to the class of linear methods and apply the same transformation to the whole image, whereas nonlinear methods can also model local geometric differences between two images.

Rigid transformations allow translation and rotation, which account for most of the expected differences between images of the same modality caused by typical experimental workflows (Fig. 4A). Affine transformation extends the geometric transformation by including the capability to scale and shear the image. Scaling becomes important when the images to register were acquired with different spatial resolutions, where one pixel in each modality represents different size areas of the sample.

The general transformation process of converting a point (pixel coordinate) in the moving image to a point (coordinate) in the fixed image can be described by

*p*′ = *f(p)* (1)

where *p,p*′∈ ℝ*^d^* represent a point in the moving and fixed space, respectively, *f* : ℝ*^d^* ↦ ℝ*^d^* is the function that transforms a point in the moving space to the fixed space and *d* is the dimensionality of the image (for a 2D image, *d = 2*). Image registration is then the estimation of the function *f* with the constraint that transformed points should correspond to the same physical location of the sample as represented by their location in the fixed image.

For linear transformations, *f* can be represented as a matrix multiplication of the form

*P_f_ = AP_m_* (2)

where *A* ∈ ℝ*^k^*^×^*^k^* is the transformation matrix, *k* = *d* + 1 and *P_f_,P_m_* ∈ ℝ*^k^*^×^*^n^* describe *n* homogeneous coordinates, for example a point in 2D space is represented as (*x,y,*1), in the fixed and moving image, respectively. Coordinates are transformed from the moving space to the fixed space by multiplication with the transformation matrix (Formula 2). If *P_f_* and *P_m_* describe matching control points selected by the user (or by any other method), it is possible to determine the transformation matrix directly by

*A = P_f_P_m_^T^(P_m_P_m_^T^)^-1^* (3)

It is important to note that, when using control point registration, the number of control point pairs determines the transformation that can be applied. This number ranges for linear methods from one (translation only), two (rigid transformation), three (affine), to four (projective). If control points are not available, then *A* (or more generally, *f*) can be estimated by maximising some measure of similarity (or, conversely, minimising some measure of dissimilarity) between the fixed and moving image, as discussed below.

Nonlinear transformations, however, cannot be represented by a simple matrix multiplication and instead are a function applied to the coordinates. These nonlinear functions are more computationally expensive than their linear counterparts, but, as mentioned above, enable tailored deformations to each part of the image, which can account for experimental artefacts such as tissue stretching or tearing (often caused by the section cutting procedure).

The second criterion by which image registration can be distinguished is the level of interactivity. Control point pairs, for example, can be either selected manually in both images, as is current practice in many MSI software tools, or extracted automatically. The latter is especially useful for nonlinear transformations, which require many matched feature coordinates distributed across the image [20].

There are also automated intensity-based image registration methods, such as phase correlation or search algorithms. These methods iteratively update the transformation matrix (within the defined geometric transformation type, e.g., affine) and compare the resulting transformed moving image and the fixed image using a similarity metric. In this iterative process, the transformation matrix is continuously updated until a stop criterion is reached, which can be that the maximum number of iterations has been reached or that the metric has converged to an optimum value (Fig. 3B).

The most appropriate similarity metric usually depends on the task and modalities involved. Commonly employed metrics are the correlation coefficient, the mean squared error, the mutual information, or derivatives of these [21]. The correlation coefficient looks at the linear relationship between two images and is, therefore, invariant to intensity offsets and different intensity scaling between the two images. This property is well suited to account for common technical variance in image acquisition from the same modality; the higher the correlation, the better the alignment. The mean squared error, in contrast, is calculated as the mean of the squared pixel-wise intensity differences and, therefore, requires the intensity values to be in the same range; the lower the value, the better the alignment. It is frequently used in template matching, where a small area of an image has to be located in a larger image [22]. For nonlinear mappings and, hence, multi-modal tasks, the mutual information has proven superior to the previously described metrics [23]. Mutal information considers the joint histogram (as an approximation for the joint probability distribution) and, therefore, does not require the images to have the same contrast mechanisms. A better alignment of two images results in a reduction of entropy in the joint histogram, which leads to higher mutual information values.

As mentioned previously, selected or pre-processed MSI images might represent intensities (selected single *m*/*z* channel or component image from dimensionality reduction) or categories (cluster or class labels as obtained from unsupervised or supervised methods, respectively). In these two different cases, different similarity metrics need to be applied when assessing the alignment quality between two images where correlation and mean squared error can only be applied in the first case and mutual information in the second.

To overcome the danger that the optimiser ends up in a local minimum, automated registration methods can be preceded by a coarse (manual) registration, include multi-resolution alignment, or employ regularisation.

### Image transformation

The actual application of the geometric transformation to the moving image is the final step in the image registration workflow. In linear transformations, this is done by matrix multiplication of the pixel coordinates of the moving image with the transformation matrix, as described previously (Formula 2). Although the pixel coordinates (*P_f_* and *P_m_*) are likely to be integer values, the values in *A* cannot be represented by natural numbers (integers) due to the inverse calculation in Formula 3. Applying Formula 2 to transform coordinates of the moving image will therefore result in coordinates that can only be represented by real numbers and are not confined to exact (integer) pixel indices (Fig. 4B). To enable assignment of intensity values to each pixel, interpolation methods are used to estimate the intensity at the (integer) pixel location based on neighbouring transformed coordinates. These can be either bilinear or trilinear for the 2D or 3D scenario, respectively, or based on the nearest transformed coordinate (Fig. 4C). In MSI, “nearest” interpolation will retain the ‘real’ pixel size of an MSI pixel in a higher resolved fixed image (Fig. 4D).

Transformations can be sequentially applied, enabling, for example, the use of an intermediary image to aid in the registration process. In the case of linear methods, a new transformation matrix, *A_combined_*, capturing two or more transformations can be created by simple matrix multiplication of the individual transformation matrices, *A_combined_ = A_n_*…*A_2_A_1_*, where *A_n_*…*A*_2_*A*_1_ ∈ ℝ*^k^*^×^*^k^* describe the *n* transformations to be applied in order from 1…*n*. Transformations with the new matrix have then no additional computational cost.

For nonlinear methods, such a simple combination is unlikely to be possible. In this case the individual transformation functions must be sequentially applied *f_n_(*…*f_2_(f_1_(P_m_))*…*)*, therefore increasing the computational burden proportional to the number of transformations being chained together.

## Existing applications and their image registration methods

Having briefly introduced the basic concepts of image registration, its methods, parameters and metrics that can be used to align two images, we review the current uses of image registration to align MSI data with clinically relevant modalities or in clinically relevant applications.

### MSI and microscopic imaging

Microscopy is routinely performed on biological and clinical tissue samples. Most commonly, H&E staining is performed to distinguish different cell types present in the sample and assess the overall architecture of the tissue (histology). In a clinical setting, this enables trained pathologists to identify the tissue’s morphology and disease state. The combination of MSI and microscopy imaging, therefore, aims to use this existing knowledge, derived from data acquired in clinical workflows, to improve the ability to extract clinically relevant information from the MSI data. Previous work has used image registration of MSI and optical images to perform histology-led MSI data acquisition [24], [25], align MSI data to external (mouse) anatomical atlases [26], [27], [28], perform MSI-led laser capture microdissection and proteomics [29], and to extract statistics from, and train classifiers for, MSI data based on histology annotations [15], [30].

An additional use of image registration of MSI and microscopy data is in the area of image fusion, a broad category of algorithms aimed at combining information from two imaging modalities, such that the combination provides more insight than each image in isolation. Various image fusion methods have been proposed to improve the spatial resolution of MSI data using information from histology microscopy images [31], [32], [33], or to incorporate signals from additional spectral modalities in multivariate analysis [34]. As such methods typically require that the images are aligned, image fusion is sometimes considered synonymous with image registration, however they are distinct fields of research and the application of one does not necessitate the application of the other.

Methods for aligning MSI and microscopy imaging data can be broadly categorised into two classes: 1) manual and semi-automated methods, requiring the user to perform one or more of the steps shown in Fig. 3 manually, or 2) fully automated methods, requiring no input from the user after the initial set-up. Manual methods have the advantage of working to the best ability of the user, but are not reproducible and can be laborious when applied to multiple samples. Automated methods, on the other hand, can be reproducibly applied to large studies with many samples, but often rely on assumptions about the data that may not hold for untested samples, so must be evaluated for each new sample type or experimental setup.

#### Manual and semi-automated alignment

Manual and semi-automated methods require user input to perform alignment of the two images. Here, we refer to manual registration as any workflow that requires the user to modify the alignment by shifting the image(s) until they are satisfied that the two images are aligned. Semi-automated methods include some automated steps, but require manual intervention capturing either domain knowledge (user selecting appropriate representative images, e.g., ion images that match the optical data) or experimental knowledge (user selecting matching fiducial markers, e.g., ablation craters). As the primary concern of this review is the alignment of MSI data, studies that use a manual step in the alignment of MSI and optical data are included here, even if additional steps are automated.

One such method, described by Verbeeck *et al*. [28], used automated methods to align experimental histology with histology from a mouse atlas, and a manual step to align MSI data with the experimental histology. By combining the two transformations, as discussed in the Section Image transformation, it was possible to use anatomical information from the mouse atlas to interpret the MSI data. The registration of MSI to histology required the user to select the most appropriate ion image, i.e., the ion image which contained features comparable to those visible in the histology data. As the sample preparation process for MSI can cause de-localisation of certain analytes (especially in the matrix application phase of MALDI or the data acquisition phase in DESI, due to the sprayed solvent), the correct choice of ion image must also take this into account. Although the authors only required a single ion image, in many situations a single ion image may not provide enough coverage of the histology image. A simple extension would be to identify and combine multiple ion images (for example as an RGB composite image). Features present in both the ion image and histology image were then used to manually define control points (also referred to as fiducial markers or landmarks), which are placed on the same feature in each modality. From the control points, it was possible to create a matrix describing an affine transform as described in the Section Image registration.

An alternative approach, proposed by Abdelmoula *et al*., automatically identified the most appropriate reference section from the atlas by selecting the image that included the hippocampus closest in size to the experimental data [26]. MSI data were aligned to the corresponding experimental histology image manually using the mass spectrometry instrument vendor’s software. The experimental histology image was then automatically aligned to the identified reference atlas image in a two-step process; affine registration of binary images of the hippocampus, followed by nonlinear registration (B-Spline) optimising mutual information.

Tian *et al*. have proposed manual intervention to the automated alignment of experimental and reference histology [35]. The inclusion of “auxiliary lines” drawn around common features in each image increased the accuracy of the resulting optical alignment and, therefore, also increased the alignment accuracy between the MSI data and the atlas by 2–5 fold, as measured by the relative overlap of expert-annotated regions.

In an alternative workflow, Patterson *et al*. and Dewez *et al*. made use of the laser craters left behind during the MALDI-MSI acquisition process to align MSI data to a post-acquisition optical image [29], [36]. Corresponding craters (central point thereof) in the optical image and pixels in the MSI data were selected manually, and an affine transformation was determined. This enabled high accuracy alignment in both cases, with errors estimated to be less than the MSI pixel size, determined either by a measure of overlap [36] or a point-to-point measure [29] (see the Section Measuring accuracy for further discussion). However, this method is limited to techniques that leave a visible post-acquisition pattern on the sample. To compensate for varying degrees of agreement for each pair of pixels in the registered datasets, Patterson *et al*. proposed to weight the MSI data, proportional to the pixel overlap, in further analysis instead of using traditional interpolation methods shown in Fig. 4 [36].

Heijs *et al*. and Patterson *et al*. used annotated optical images to select specific regions on the sample to analyse by MSI [24], [25]. Both methods employed automated optical-to-optical registration enabling the annotations to be defined by neighbouring tissue sections (which were, for example, stained using H&E) to those analysed by MSI. However, the final alignment of the annotated optical image to the instrument coordinate system (and, therefore, to the MSI data) is performed in the instrument vendor’s software, by manually adjusting the position of the images until the user is satisfied with the result.

#### Automated alignment

The primary challenges in automated alignment of MSI and optical images are that the contrast mechanisms of the two techniques are different and there is often a large discrepancy between the pixel sizes of the two images. This means that certain registration methods and metrics are not suitable, as discussed in the Section Image registration.

One way to align different modality data is to convert both images to a binary mask (highlighting the same feature) and register the binary images (as illustrated in Fig. 3B). Anyz *et al*. used the whole tissue area as the feature to align, and so generated binary masks of the tissue in LA-ICP-MS data and the microscopic image acquired on serial sections (further details not provided), which were used to determine an affine transformation matrix by minimising the sum of squared differences [30]. This approach to aligning data from multiple sources, while simple and easy to implement, has some limitations. If there is any rotational symmetry in either mask, then there will be multiple ‘best’ transforms that could be found. Any features within the tissue are ignored; therefore, if the masks include any inaccuracy in the shape of the tissues then the resulting alignment will be poor. For example, if the so-called ‘halo effect’ in MSI (the detection of biological compounds around the edge of the tissue) is included in the binary mask (which is a realistic scenario, as it can be difficult to determine the exact edge of a tissue when a ‘halo’ is present) then the size of the MSI tissue will be overestimated. Consequently, the resulting transformation will compress the MSI data into the centre of the histology tissue, causing larger errors closer to the tissue edge.

A more sophisticated method was proposed by Abdelmoula *et al*. [14]. MSI data were processed to remove background pixels (non-tissue related regions) and then reduced using t-distributed stochastic neighbourhood embedding (t-SNE), a nonlinear dimensionality reduction method, to three dimensions that were then encoded in an RGB image, before being further compressed to a greyscale image (one dimension). Histology data were processed to exclude background regions and converted to greyscale. This then enabled the use of existing tools (such as the general image registration toolbox, elastix [37]) to align the two greyscale images by optimising mutual information (as the images originated from different modalities). This method can be performed on any MSI modality as there is no requirement of experiment-specific features (such as laser ablation craters) to be present. However, the dimensionality reduction step must extract features comparable to those present within the histology. While this is likely to occur due to the assumption that differently stained regions in the histology are chemically different and MSI readily detects chemical differences, there is no guarantee and so the method must be evaluated on a sample-by-sample basis.

Despite this potential limitation, this method has inspired a number of further works, which have built upon it and expanded its use cases. Škrášková *et al*. combined the automated alignment to the Allen brain atlas, proposed by Abdelmoula *et al*. [26], and the method described previously to enable automatic alignment of SIMS data to the Allen Mouse Brain Atlas [27]. More recently, Race *et al*. proposed a modified workflow using deep learning to classify the histology data (to provide more comparable features to those visualised by MSI) and Uniform Manifold Approximation and Projection (UMAP) as the dimensionality reduction technique to process the MSI data (instead of t-SNE), reducing execution time considerably [15]. It was also shown that direct reduction to a single dimension using UMAP reliably retained more features than the original two-step dimensionality reduction process proposed by Abdelmoula *et al*. Using the Hausdorff distance (see the Section Measuring accuracy), Race *et al*. demonstrated a worst-case registration accuracy approaching the pixel size of the MSI data [15].

### Multiple MSI datasets

Mass spectrometry imaging datasets can also be spatially registered to each other. There are two scenarios where this is necessary: 1) in 3D-MSI where consecutive sections are aligned to form a volumetric representation of the sample, and 2) to align multi-parametric MSI datasets from consecutive, or the same, sections to combine different molecular information per pixel.

#### Multiparametric MSI

The idea of spatially aligning multiple MSI datasets is based on the fact that the attainable information in an MSI experiment strongly depends on the parameterisation of the experiment, e.g., the type of matrix for MALDI-MSI, the solvent for DESI-MSI, the ion gun for SIMS, the polarity, or even the ionisation source choice itself. These choices are largely exclusive for a certain molecular class. In order to increase the information on a per pixel level, some works have performed dual polarity lipid MSI experiments in two runs, but with spatial offsets in *x* and *y*, which allowed the creation of virtually larger pixels that contained the data from both polarities [38], [39]. As the two experiments were performed consecutively without removing the sample from the mass spectrometer, no image registration was necessary.

Conversely, if the section is removed from the mass spectrometer, or another section is used, image registration becomes necessary. This has been shown by Patterson *et al*. who developed an image registration and data analysis workflow that allows combining data from MSI experiments with incompatible sample preparations [7]. This way the authors could combine the data obtained in five different experiments on a per pixel level from proteins, dual polarity phospholipids, cholesterol, free fatty acids, and triglycerides. While this allowed finding spatially correlated ions across the different molecular classes, the integration of these datasets for multivariate algorithms requires new normalisation strategies to guarantee equal contributions.

#### 3D-MSI

Aligning MSI datasets spatially to each other is also necessary in cross-section based three-dimensional (3D) MSI. This is mainly encountered in MALDI and DESI-MSI where serial sections of the sample are used to obtain a volumetric representation of the sample [40]. While the alignment of a stack of 2D images to a 3D volume shares similarities to the previous approaches, the overall aim is to reconstruct the original 3D volume as close to the original *in vivo* sample as possible.

**Fig. 5:**
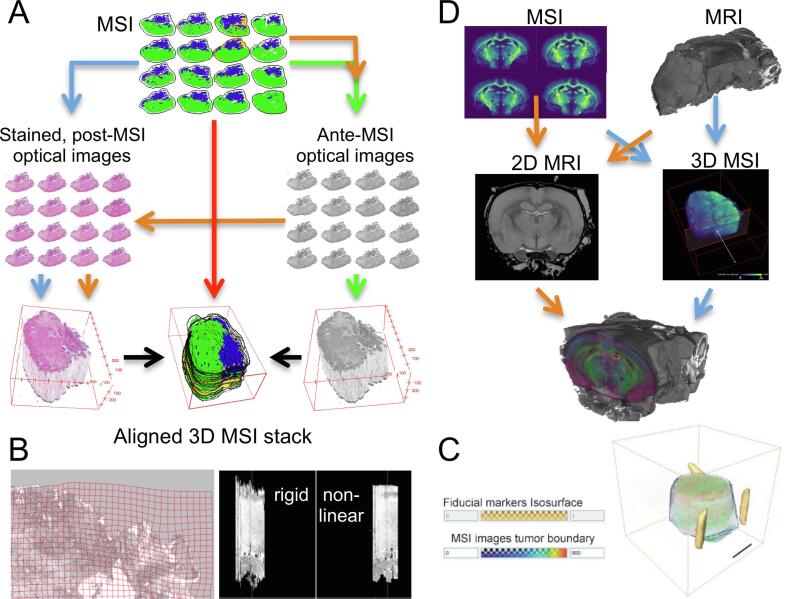
(A) Different registration workflows, depicted as green, orange, blue, and red paths, to reconstruct volumes from 3D mass spectrometry imaging (MSI) data. Since optical images usually have a higher spatial resolution than MSI images and are often already experimentally linked to the MSI data, most stack alignment workflows base their reconstruction on the alignment of the optical images first. These optical images can be obtained before (ante-) or after (post-) the MSI experiments. (B) The alignment quality of nonlinear transformations can be visually evaluated using transformed grids to exclude over-registration effects in 2D (left) and orthogonal slicing can be used to evaluate the overall aligned stack (right). Adapted with permission from [43]. Copyright 2016 Elsevier B.V. (C) To avoid the “banana-problem” during volumetric reconstruction, i.e., the straightening of curved volumes, three-dimensional fiducial markers can be introduced next to the embedded tissue sample which serve as reference for a correct volumetric reconstruction. Adapted with permission from [44]. Copyright 2012 American Chemical Society. (D) Registration workflows for aligning MSI with MRI, by constructing a 3D volume from 2D-MSI data and aligning the 3D-MSI and 3D-MRI volumes (blue arrows) or by selecting and registering 2D slices from the MRI volume which correspond to the same location as the acquired 2D-MSI data (orange arrows). MRI data from the Waxholm Space atlas of the Sprague Dawley rat brain [45]. Abbreviations used: mass spectrometry imaging, MSI; magnetic resonance imaging, MRI.

To achieve this, several workflows have been developed over the last 16 years, which primarily differ in the actual images that are used to align the stack of 2D images to a 3D volume (Fig. 5A). The first section-based 3D-MSI study was conducted in 2005 by Crecelius *et al*., who were interested in the 3D visualisation of protein signals in mouse brain [41]. Since the authors found that “the cumulative error produced by aligning successive ion images to be greater than that produced by aligning each ion image to its respective optical image”, the alignment between the serial sections is based on registering optical images taken prior to the MSI experiment (Fig. 5A, green workflow). Several methodological studies followed that used, adapted, or improved this workflow by, for example, aligning the 3D stack based on the stained microscopic images (Fig. 5A, orange workflow) [8], [42]. Lotz *et al*., for instance, added to the initial rigid registration a second, nonlinear registration for refinement of the stack registration of the optical ante-MSI images [43]. The registration quality was evaluated visually through orthogonal slicing (Fig. 5B, right) and by visualising the geometric transformations using grids (Fig. 5B, left), both of which validated their approach.

Other registration workflows for 3D-MSI have been developed. Patterson *et al*. demonstrated how open source software can be used to make and investigate 3D-MSI reconstructions of several atherosclerotic plaques [39]. While it shares the above approach for reconstructing the 3D model based on stained optical images using rigid-body registration, the 2D-MSI datasets were directly aligned to their corresponding H&E stained images without any other intermediate optical image (Fig. 5A, blue workflow). This in turn required individualised pre-processing strategies for all H&E images, including removal of background, segmentation, and noise removal.

To complete the set of 3D alignment approaches in MSI, Dueñas *et al*. directly co-registered 62 consecutive cross-sectional MALDI images to study lipid distributions in single celled zebrafish zygotes. (Fig. 5A, red workflow) [46]. Given the strict spherical shape of the cell, translation and rotation were used to manually place the 2D ion images on top of each other in the TrakEM2 module of ImageJ. Since this study was performed at high-resolution with 10 μm pixel sizes, the rationale to use the optical images due to their higher resolution for the 3D stack alignment is no longer as relevant as it was at the beginning of 3D-MSI in 2005.

Given this new situation, Cordes *et al*. recently developed the M^2^aia tool for the reconstruction of 3D-MSI datasets in a mostly automated way. It uses structurally rich MSI images, which have to be pre-selected by the user, to apply rigid and deformable image-based registration of consecutive MSI slices to create volumetric MSI datasets (Fig. 5A, red workflow) [47]. This tool has also recently been employed by Enzlein *et al*. for creating 3D-models of amyloid plaques as measured by MALDI-MSI with voxel sizes of 20 × 20 × 10 μm [48].

While the above-mentioned studies have provided satisfactory results, some important issues remain in the reconstruction of volumetric shapes based on a stack of cross-sections. One is the so-called “banana problem” which describes the difficulty to reconstruct a 3D-curved object from cross-sections without any additional information [49]. In order to address this challenge, additional information can be added in the form of fiducials.

Chughtai *et al*. were the first to propose the insertion of fiducial marker compounds into the embedding matrix of tissues [44]. The authors injected various liquid compounds into gelatine blocks in proximity to the embedded breast cancer xenograft tissue. These markers could be detected by both MSI and optical microscopy, which improved the reconstruction of 3D-curved volumetric models in both modalities (Fig. 5C). In this study, the images of the 2-mm thick cross sections were manually overlaid in GIMP 2.6.

Subsequent work by Anderson *et al*. improved the idea of embedded markers and, instead of liquid dyes, proposed the insertion of multi-modal compatible rigid rods into the embedding matrix of the sample [50]. To optimally address the banana-problem, the rods were arranged into the block around the sample tissue in a triangular fashion, which describes the tissue space best in all three dimensions. This approach was applied to optic nerve tissues from control and glioma-bearing mice, which were both embedded in the same block. Thirty-three individual 2D MSI data sets were acquired and co-registered following the green workflow (Fig. 5A).

As a last point of discussion, most of the 3D-MSI studies perform volumetric reconstruction with the argument that “the true molecular distribution cannot be adequately assessed when only one or a few sections are analysed”, as stated by Anderson *et al*. [50]. While this is certainly true, it is not an adequate rationale for the necessity of a 3D-MSI volumetric reconstruction, but rather for the more comprehensive description of a sample. The latter can be equally well realised by visualising and analysing the sections side-by-side, unless the (*x*,*y*,*z*) positions directly affect the data analysis, as demonstrated by Inglese *et al*. [18] and Enzlein *et al*. [48].

3D volumetric reconstruction is, however, helpful, or even vital, when integrating MSI data with data from *in vivo* technologies.

### MSI and *in vivo* techniques

The ability to link the detailed chemical information provided by MSI to non-invasive, *in vivo* techniques, such as MRI, CT and PET harbours great potential for diagnosis and personalised medicine due to their high complementarity. As MRI, CT and PET typically produce 3D images, there are two general approaches to the problem of aligning *in vivo* and MSI data. The first is to turn it into a 2D registration problem (see Fig. 5D orange arrows), by selecting the section from the *in vivo* data that most closely matches the MSI data. The second requires 3D-MSI data to be acquired and aligned using techniques such as those described in the previous section to produce a 3D volume (see Fig. 5D blue arrows), which can then be aligned to the 3D *in vivo* volume. The latter being the more challenging of the two approaches. Using one of these two approaches, most studies to date have focused on the integration of MSI and MRI data.

Sinha *et al*. presented the first example of combining MSI data with MRI images to integrate proteomic profiles and *in vivo* data from a whole mouse head [51]. MRI data were acquired immediately prior to sacrifice of the mouse, which was subsequently perfused with saline and frozen in an ice block. During the sectioning process, high-resolution optical images were acquired of the block face prior to each cut. These images were then reconstructed into a 3D volume by sequentially performing 2D registration, using the previously registered section as the ‘fixed’ image and the next section in the series as the ‘moving’ image, to find a translation and (in-plane) rotation transformation which optimised normalised mutual information (orange arrows, Fig. 5D). MSI images were then registered to their corresponding (registered) blockface image using the iterative closest point (ICP) algorithm, which aligned manually highlighted features (contours) in the images (e.g., outline of brain and head). Finally, the blockface image volume was aligned to the MSI data by finding the rigid transformation which optimised normalised mutual information.

Oetjen *et al.* combined 3D-MSI data and MRI data of a mouse kidney [52]. The individual MSI images were first aligned to one another using rigid registration, as originally performed by Trede *et al.* [8], followed by elastic registration to compensate for local deformations caused by sectioning. The resulting 3D volume was then aligned to MRI data using MeVisLab (blue arrows, Fig. 5D), however further details of this key step (and the elastic registration) were unfortunately omitted.

Extending their work on registering MSI data to the Allen Brain Atlas, Verbeeck *et al*. presented a workflow for aligning MSI data to an MRI atlas, the Waxholm Space atlas of the Sprague Dawley rat brain [45]. The appropriate MRI section from the atlas (determined by relative proximity in the brain to the section imaged by MSI) was manually extracted. PCA was used to create a low dimensional representation of the data, and the resulting score image, which most closely matched the distribution visible in corresponding MRI atlas section, was manually identified and selected for use in the registration process. Non-rigid (free form deformation) registration was then performed to align the two images, using squared correlation coefficient as a similarity measure.

In a similar vein, Abdelmoula *et al*. extended their own work on registering MSI data to the Allen Brain Atlas, presenting a workflow for the alignment of MSI and MRI data [10]. In this work, a representative image for each MSI image (each section) was created, using t-SNE to reduce the dimensionality of the data to three. These data were then encoded in the CIE L*a*b* colour space. As each dataset was processed individually, there can be no guarantee that the same features will be represented in the same way in the low dimensional space and, therefore, no guarentee that they will be highlighted in the same colour in the representative images of each section. Each representative MSI image was then registered to the corresponding MRI slice (selected manually based on visual similarity) by first optimising an affine transformation for the global positioning, followed by a BSpline transform capturing nonlinear deformations, by optimising mutual information.

In comparison to the integration of MSI and MRI data, the integration of MSI and CT is relatively unexplored. Schioppa *et al.* demonstrated a proof of principle workflow by manually aligning data acquired from a fish head [53].

## Challenges

Despite the strides the community has made in enabling integration of MSI data with clinically relevant techniques, there remain a number of key challenges to address.

### Method evaluation and comparison

As seen throughout this review, there are many workflows for each of the application areas discussed, but no comparison exists. There are two aspects preventing this: (1) the lack of reproducibility of methods, and (2) the lack of reported registration quality.

Reproducibility remains a significant issue due to the absence of published code, software, and data. A list of existing software tools (and their capabilities and target audience) is included in Table 1. The software tools that provide a user interface and target a wide audience (that may not have programming skills), implement interactive, manual methods, relying on the user to accurately align the data. The automated methods discussed in this review can be applied to data loaded in extensible software, such as SpectralAnalysis, Cardinal or rMSI, but require the user to first re-implement the algorithm, which is a time consuming process that is also difficult to validate, as the data used to describe the algorithm is often unpublished. Moreover, since most of the publications have been published in analytical journals, the methodological descriptions were primarily focused on aspects other than the registration. This has also led, for the sake of simplicity and to match the readers’ interest, to omitting important technical details of the image processing workflow making an exact re-implementation impossible.

**Table 1:** Software used in mass spectrometry imaging for image registration.

**Software name**	**Vendor**	**User interaction**	**Capabilities**	**User type**
LipostarMSI	Molecular Discovery	Interactive overlay	Rigid + scaling	MSI-expert
HDI	Waters	Interactive overlay	Rigid + scaling	MSI-expert
FlexImaging	Bruker Daltonics	Based on control point selection	Affine	MSI-expert
SCiLS Lab	Bruker Daltonics	Interactive overlay assisted by control point selection	Rigid + scaling	MSI-expert
MSIReader [55]	free	Interactive overlay	Rigid + scaling	MSI-expert
M^2^aia [47]	free	Interactive overlay with access to elastix parameter file	Linear and non-linear registrations	MSI-expert, Image-analysis expert
ImageJ, FIJI	free	Graphical user interface	Linear and non-linear registrations	MSI-expert, Image-analysis expert
SpectralAnalysis [56]	free	Matlab routines from image processing toolbox		Expert
R packages for MSI such as Cardinal [57], MALDIquant [58] and rMSI [59]	free	R packages for image registration		Expert
Open-source toolkits such as ITK or elastix	free	Command line	Linear and non-linear registrations	Expert
RegCombIMS[7]	free	R	Linear and non-linear registrations	Expert

**Fig. 6:**
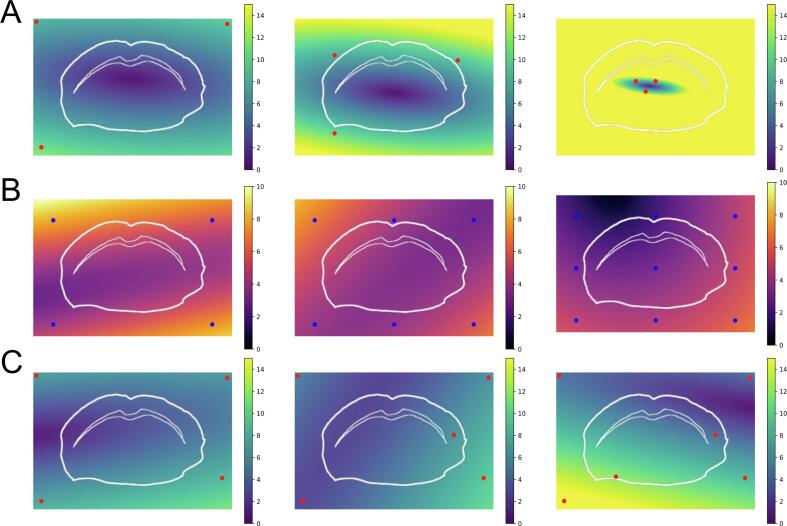
Simulation of registration errors when using different strategies for selecting control points. Actual errors will depend on the real transformation, and so the values here should only be used as an illustration. (A) Fiducial markers (in red) placed away from tissue (left, mean error 3.36), close to tissue (middle, mean error 5.21), and concentrated in a small area (e.g., based on localised anatomy) (right, mean error 38.83). (B) Control points (in blue) placed in a grid of 4 points (left, mean error 4.06), 6 points (middle, mean error 3.68) and 9 points (right, mean error 3.13). (C) Arbitrary placement of four control points (left, mean error 3.88), five control points (middle, mean error 3.88) and six control points (right, mean error 6.89).

This also impedes *meta*-studies that compare the different approaches in order to score and optimise registration workflows, as previously performed for other experimental workflows in MSI [54]. It is clear that these initiatives and comparisons need to account for the study-specific experimental setups, requirements, and goals, which can only be guaranteed if the original data is accessible. However, they also must consider non-deterministic aspects of the registration workflows, as some methods require user interaction to varying degrees ranging from manually performing the registration to manually selecting features for automatic alignment (Fig. 6).

Another factor hindering the comparison of the different approaches is the lack of reported registration accuracy by most studies. This is the result of: 1) recognition of the necessity to determine and report the accuracy, and 2) the difficulty and expertise in doing that appropriately. With respect to the first point, most MSI studies are performed with pixel sizes equal to, or larger than, the error of current non-sophisticated registration workflows, hence, there is no apparent need to improve anything. The second point refers to the fact that the evaluation of the registration accuracy is non-trivial and requires image analysis expertise. However, even if the registration accuracy is evaluated, there is no standard, agreed upon methodology and different metrics have been used that are not directly comparable. While the aim of such comparison initiatives would clearly be to improve registration accuracy and precision, a key milestone would be to propose, and perhaps establish, standard evaluation methods in the field of MSI with a set of appropriate metrics.

### Improving and measuring accuracy

#### Improving accuracy

With the increase in spatial resolution in MSI approaching cellular resolution, there is an increased need for accurate registration methods. Improving the accuracy of alignment between multi-modal images, such as those reviewed here, is an active area of research.

Currently, most registration of MSI and microscopic images is performed using manual control point registration. A crucial aspect on the registration accuracy in this approach is the correct selection of the landmarks, in terms of spatial distribution and number of control points. Generally, it can be said that the *more* markers and the more spread *around* the tissue, the lower the registration error (Fig. 6). The necessary human intervention limits both aspects, since a human can only select a limited number of control points and the average MSI users are not aware of the optimal placement of the landmarks.

Automated control point extraction tools, such as SURF, which are successfully used in other fields of biological multi-modal imaging, are able to extract hundreds of matching features [60]. However, the results strongly depend on pre-processing of the images, which in turn might require user intervention and expertise [61].

Feature extraction is not only limited to the detection of matching points between fixed and moving images. Automated feature extraction can also rely on, for example, the selection of an appropriate channel or on dimensionality reduction methods (Fig. 3), such as PCA used by Trede *et al*. to create volumetric 3D-MSI datasets [8]. Such a registration approach relies on the assumption that the reduced/selected data will highlight features comparable to that in the other modality. When the second modality is the same as the first (as in 3D-MSI), this is a reasonable assumption, however when the second modality has completely different contrast mechanisms, such as histology and microscopy (as described in the Section MSI and microscopic imaging), such an assumption is not guaranteed. One way to improve automated methods may be to make use of previous knowledge in the form of trained classifiers to aid in the feature extraction process. For instance, classifiers trained to identify features visible due to the contrast mechanisms of the second modality (e.g. anatomical regions from microscopy data) could be used to automatically extract ion images from the MSI data, which visualise the same features.

Another way to improve the accuracy could be by the use of nonlinear registrations, as already evidenced by some MSI-related studies (Fig. 5B) [7], [24], [43], [48]. While the application of these algorithms carries huge potential, some studies found that the use of nonlinear registration with default parameterisation are too erroneous for clinical use [62]. For this reason, nonlinear registration is usually preceded by several linear pre-alignment steps to assist the optimiser function in finding the global optimum [47], [63].

In line with this, we believe the multi-step registration approaches carry huge potential in MSI; for example, simple manual registration by the MSI-experimenter assisted by an automated optimisation using more sophisticated registration algorithms [16]. The recent M^2^aia software exemplifies this by allowing an interactive pre-alignment of the 3D-MSI stacks before the application of automated linear and nonlinear registrations [47].

Still, 3D-MSI could benefit from improvements related to registration. Current reference-free approaches suffer from the potential to create different errors, such as the banana-problem [49] or the propagation and accumulation of registration errors through the 3D stack [64]. While there have been attempts in the MSI community to address these by the introduction of spatial reference objects [44], [50], the latter error occurs because the registrations are performed sequentially in pairs and with a directionality. Global optimisation approaches exist that optimise the alignment of more than two images either based on parallel algorithms, which couple all local neighbourhood transformations into a system of equations [65], or by modelling stack misalignment by using heat diffusion equations [64].

All of these approaches require extensive expertise in the field of image analysis and pre-processing, which is not available in every MSI laboratory. We, therefore, see a need for the development of simple-to-use MSI image registration software that makes use of advanced image registration procedures. This will increase the overall registration accuracy of the MSI community. These processes must be generic enough to account for the varying experimental methods and workflows.

#### Measuring accuracy

**Fig. 7:**
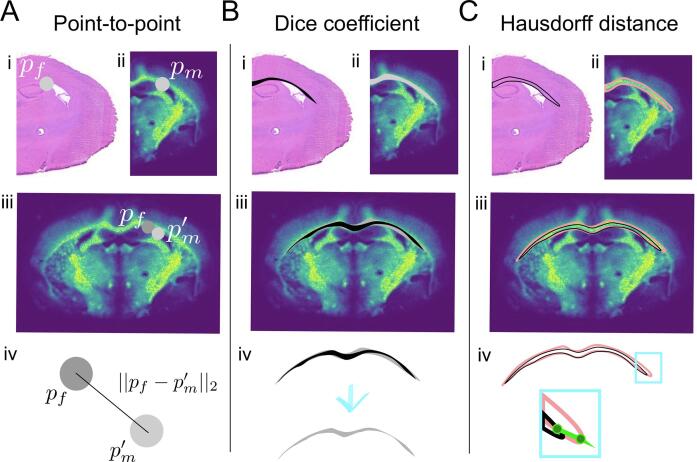
Methods used for measuring the alignment accuracy. (A) Point-to-point. Comparable points are marked on each image (e.g., the edge or middle of clear anatomical structure, i and ii), the points in the moving are transformed to the fixed image (iii) and the (Euclidean) distance is measured (iv). (B) Dice coefficient. Comparable regions are marked in each image (for example, drawing around anatomical structures, i and ii), the region in the moving image is transformed to the fixed image (iii) and the proportional overlap of the two shapes is calculated (iv). (C) Hausdorff distance. Regions are marked and transformed (i–iii) as in (B), where the longest (marked in green) of all shortest possible paths from one region to the other is then determined (iv).

There is no consensus within the community on how accuracy should be measured and reported. At least three different methods have been used in the studies covered here: (i) point-to-point comparison [29], (ii) the Dice coefficient, [30] and (iii) Hausdorff distance [15]. The methods are summarised pictorially in Fig. 7. Point-to-point comparison requires an expert to label corresponding points in the two images (based on features, for example the same exact point in the *corpus callosum* of a mammalian brain). The distance between the points in the fixed image and the transformed points from the moving image are then compared, and the Euclidean distance is calculated (Fig. 7A). This gives an accurate measure of the accuracy of single pixels (points) within the image, but limited information for larger features, unless repeated across the image. This feature is also available in the recent M^2^aia software [47].

The Dice coefficient measures the degree of overlap of two shapes (Fig. 7B). These shapes could be defined manually (expert draws around the same feature in both images) or automatically via classification or clustering. The degree of overlap is then calculated between the shape in the fixed image and the transformed shape from the moving image. This provides a measure of goodness of fit for the selected features.

Finally, the Hausdorff distance is calculated by first determining the shortest distance from every point on the polygon (shape, as in the Dice coefficient) in the fixed image to the transformed moving image polygon (Fig. 7C). Then the longest distance of all these distances is taken. Similar to the Dice coefficient, the polygons can be defined by an expert, or automatically by classification and clustering. This gives a measure of the worst-case accuracy for the selected features.

As each metric measures slightly different aspects of the alignment, it is not possible to compare them directly, nor is it possible to select one single metric that is most suitable in all cases. A combination of different metrics is, therefore, the most accurate way to describe registration error. Moreover, as can be recognised in Fig. 6, the registration error is characterised by spatial variance, which makes it necessary to measure the error at different positions in the image. It is also worth noting, that the input used to calculate the accuracy in each case is typically provided manually (points or shapes) and, hence, is subject to expert bias. Evaluating, even manually, the error at several positions provides a more thorough understanding of the registration error [16].

## Outlook

To help address some of the challenges listed above, we propose a roadmap, which aims to improve reporting, reproducibility and facilitate comparisons and the development of new algorithms, software and tools.

First, we propose an extension of the current imzML format (open format for storing MSI data [66]) to include details of linked data (e.g., optical images), the transformation and parameters for aligning the data, and methods used to determine the transform. This will facilitate sharing of more complete datasets for multi-modal studies, as well as allowing data to be aligned in one tool, and visualised and further processed in another.

Following the above guidance, we propose providing the community with a collection of publicly available benchmark datasets, similar in style to that gathered by Oetjen *et al*. [67], but for registration purposes. This benchmark dataset shall ideally include data covering different sample types, ionisation sources, and second modalities. The availability of a community benchmark dataset is hoped to attract image analysis expertise from outside the field of mass spectrometry imaging to apply existing, or develop new, image registration methods for mass spectrometry imaging-specific challenges; including appropriate criteria to evaluate registration quality. This will also enable evaluation of new and existing methods on a wider array of experimental setups, and, in combination with the extended imzML capturing transform details, enable direct comparison of existing results. To achieve this, an open collaborative initiative has been started (“MSI&Co”), which aims to address these challenges (https://github.com/MSIandCo/MSIandCo).

In conclusion, the measures specified in this proposed registration roadmap for the field of mass spectrometry imaging have the potential to surmount the current bottleneck in image registration, which will ultimately boost future multimodal applications. This is expected to facilitate exploitation of the combined imaging technologies and, therefore, augment applications of MSI.

## Declaration of Competing Interests

The authors declare that they have no known competing financial interests or personal relationships that could have appeared to influence the work reported in this paper.
